# Effect of inoculation method on the determination of decontamination efficacy against *Bacillus* spores

**DOI:** 10.1007/s11274-014-1684-2

**Published:** 2014-06-14

**Authors:** Shawn P. Ryan, Sang Don Lee, M. Worth Calfee, Joseph P. Wood, Stella McDonald, Matt Clayton, Nicole Griffin-Gatchalian, Abderrahmane Touati, Luther Smith, Melissa Nysewander

**Affiliations:** 1 US EPA, Office of Research and Development, National Homeland Security Research Center, MD E343-06; 109 TW Alexander Dr., Research Triangle Park, NC 27711 USA; 2 Arcadis Geraghty and Miller, Inc., Durham, NC USA; 3 Alion Science and Technology, Inc., Durham, NC USA; 4 Formerly Alion Science and Technology, Inc., Durham, NC USA

**Keywords:** Decontamination, Sporicide, Anthrax, *Bacillusanthracis*

## Abstract

Decontamination studies investigating the effectiveness of products and processes for the inactivation of *Bacillus* species spores have traditionally utilized metering viable spores in a liquid suspension onto test materials (coupons). The current study addresses the representativeness of studies using this type of inoculation method compared to when coupons are dosed with a metered amount of aerosolized spores. The understanding of this comparability is important in order to assess the representativeness of such laboratory-based testing when deciding upon decontamination options for use against *Bacillus anthracis* spores. Temporal inactivation of *B. anthracis* surrogate (*B. subtilis*) spores on representative materials using fumigation with chlorine dioxide, spraying of a pH-adjusted bleach solution, or immersion in the solution was investigated as a function of inoculation method (liquid suspension or aerosol dosing). Results indicated that effectiveness, measured as log reduction, was statistically significantly lower when liquid inoculation was used for some material and decontaminant combinations. Differences were mostly noted for the materials observed to be more difficult to decontaminate (i.e., wood and carpet). Significant differences in measured effectiveness were also noted to be a function of the pH-adjusted bleach application method used in the testing (spray or immersion). Based upon this work and the cited literature, it is clear that inoculation method, decontaminant application method, and handling of non-detects (i.e., or detection limits) can have an impact on the sporicidal efficacy measurements.

## Introduction

The release of *Bacillus anthracis* spores from envelopes mailed through the U.S. Postal Service (USPS) in 2001 (henceforth, Amerithrax) resulted in the first bioterrorism-related anthrax cases in the U.S. (Jernigan et al. 2001) Twenty-three facilities were confirmed contaminated to at least some degree (Sharp and Roberts 2006). In total, remediation occurred over several years (Sharp and Roberts 2006) and decontamination costs alone (not overall remediation costs) were estimated to have exceeded $290 million (Schmitt and Zacchia 2012).

At the time of the 2001 incident, the need to decontaminate entire public facilities for bioweapons was unprecedented. No decontamination products or technologies were registered by the EPA under the Federal Insecticide, Fungicide and Rodenticide Act (FIFRA), and hence proven, for use against *B. anthracis* spores (Martin 2003; Canter 2003). Through on-site trials and vendor-supplied data, products and application conditions for successful remediation of the facilities were ultimately selected and employed. The techniques used, often in combination, included several liquids with claimed sporicidal efficacy, fumigants, and removal and off-site treatment of equipment and materials (Sharp and Roberts 2006). During the overall remediation efforts, considerable expertise was gained, but review of these efforts concluded that improved methods were needed for effective remediation following contamination with *B. anthracis* spores (Whitney et al. 2003).

One important data gap highlighted from a review of the Amerithrax incident response was that laboratory data generated for the assessment of sterilants or disinfectants for *B. anthracis* were difficult to interpret relative to the specific application needs (Whitney et al. 2003). In a review of data published from 1930 to 2002 on the chemical inactivation of *B. anthracis* spores, Spotts Whitney et al. (2003) reported on several difficulties associated with the homeland security application. One highlighted difficulty was that results from laboratory experiments do not specifically address questions regarding the best methods for inactivating spores on commonly encountered materials such as mail, carpet, and other porous objects. A second gap was understanding the relationship between sporicidal efficacy testing in the laboratory and use in field-level decontamination.

With regard to efficacy testing, the EPA’s regulatory standard for performance testing of sporicides (for registration under FIFRA) remains the Association of Official Analytical Chemists (AOAC^®^ International) Official Method™ 966.04, Sporicidal Activity of Disinfectants (AOAC International 2006). The AOAC^®^ International test method also has importance to the U.S. Food and Drug Administration’s approval process for chemical sterilants related to sterilization of medical equipment (Tomasino et al. 2008). However, data generated from Official Method™ 966.04 are more relevant to clinical settings than decontamination of buildings and environmental surfaces (Tomasino et al. 2008; AOAC International 2008). Based upon the recognized needs, as highlighted in Spotts Whitney et al. (2003), EPA has developed additional quantitative test methods to determine the efficacy of sporicidal decontaminants on surfaces relevant to field-use (Ryan et al. 2010). Currently, demonstration of a >6-log inactivation of *B. anthracis* or an appropriate surrogate spore (e.g., *B. subtilis*) using a quantitative test method, such as AOAC^®^ International Method 2008.05 (AOAC International 2008), ASTM 2197-02 (ASTM International 2002), or ASTM 2414-05 (ASTM International 2005), by a decontaminant is being considered as a requirement for product registration as a sporicidal agent against spores of *B. anthracis* (U.S. Environmental Protection Agency FIFRA SAP Meeting No. 2007-50 2007).

Considerable data have been generated using the methods cited above or similar, acceptable, quantitative methods regarding product efficacy against *B. anthracis* spores on complex material surfaces, such as those found in facilities and outdoors (U.S. EPA 2011b; Wood et al. 2011; Calfee et al. 2011; U.S. EPA 2007, 2010; Wood 2009; Rogers et al. 2005, 2006, 2007; Ryan et al. 2012; Calfee 2012; Rastogi et al. 2010). These results helped bridge a critical knowledge gap with respect to relevant product efficacy. However, as summarized by Ryan et al. (2010) all of these methods are carrier-based in which a piece of test material is inoculated with a spore suspension. An advantage of liquid inoculation is accuracy and precision in the application of the target organism onto the materials; i.e., a predetermined and highly repeatable amount of an organism can be applied to carriers. However, during the Amerithrax incident, surfaces were contaminated with a dry powder (Weis et al. 2002). While there are many advantages to the use of liquid inoculation, the correlation between such testing and the decontamination of aerosol-deposited biological organisms (primarily dried spores) remains undetermined. Potential clumping, penetration of the liquid within materials, and layering during the physical drying process on non-porous materials (i.e., outer edge of the droplet becoming higher in concentration of the organism as the spot dries) suggest that biological organisms deposited as liquids may be more difficult to inactivate on some surfaces than the same organisms deposited as aerosols (Sansoë-bourget 2006). Along the same lines, Edmonds et al. (Edmonds et al. 2009) reported a difference in recovery when sampling liquid- and aerosol-deposited spores.

The focus of the current study was to assess the impact of the inoculation method on the determination of sporicidal efficacy determined at various contact times related to *Bacillus* spores. Two decontaminants were chosen for this effort, chlorine dioxide gas and pH-adjusted bleach. These decontaminants were chosen based upon the breadth of existing data utilizing these chemicals, their effectiveness, and likelihood of being used in the case of future incidents (Ryan et al. 2010). These data further our understanding of the relationship between laboratory-determined efficacy via traditional methods and anticipated effectiveness of products in field applications.

## Materials and methods

For this study, uniform pieces of building materials (henceforth, referred to as coupons) were inoculated with spores of *Bacillus subtilis* and treated using one of three decontamination procedures. Decontamination efficacy was determined based upon the comparison of the number of spores [measured as colony forming units (CFUs)] recovered from coupons not exposed to decontamination versus the recovered CFUs from decontaminated coupons. Temporal differences in decontamination efficacy were used to elucidate the impact of the inoculation method as a function of coupon material type and decontamination procedure. The test matrix included a variation of the latter two parameters (material type and decontamination procedure) in order to ensure that the impact could be as broadly understood as possible, within the bounds of the study. This section discusses the spore preparation, coupon preparation, decontamination methods, sample analysis protocols, and statistical data analysis methodology.

### Spore preparation

The *Bacillus subtilis* spores (ATCC 19659, Manassas VA) were prepared as previously reported by Rastogi et al. (2009), Rogers et al. (2005) and further described in Lee et al. (2011). Briefly, a seed culture was initiated in tryptic soy broth (TSB) and then sporulated on sporulation media at 37 °C for 10 ± 4 days. The sporulation media was a mixture of 23 g Lab Lemko (Oxoid Ltd., Hampshire, UK) agar, 2 g tryptone (Fisher Scientific, Fair Lawn, NJ), 23 g yeast extract (Becton–Dickinson, Sparks, MD), 1 % MnCl_2_ (Sigma Chemical Co., St. Louis, MO) and 2 g agar (Becton–Dickinson, Sparks, MD). Microscopy (BX50, Olympus^®^, Miami, FL) was used to determine at least 90 % of the cells had sporulated. The spores were then harvested and triple washed (centrifugation followed by resuspension with chilled deionized water). The resulting spore preparation was reconstituted in chilled deionized water and heat-shocked at 65 °C for 30 min.

For use in aerosol inoculation of the coupons, the spore preparations were then loaded into metered dose inhalers (MDIs) by the aerosol science laboratory at Edgewood Chemical and Biological Center (Aberdeen Proving Ground, MD) as described in Carrera et al. (2005). Each MDI contained *Bacillus subtilis* spores in an ethanol (analytical reagent grade, Mallinckrodt Inc., Paris, KY) solution and propellant. The spore concentration in an MDI was 0.5 or 0.05 % w/w (resulting in approximately 10^8^ and 10^9^ CFU per MDI actuation, respectively).

### Material coupon preparation

The material types used in this study were chosen based upon results from previously published decontamination studies (U.S. EPA 2011b; Wood et al. 2011; Calfee et al. 2011; Rogers et al. 2005, 2006, 2007; U.S. EPA 2007, 2010; Wood 2009; Ryan et al. 2012; Calfee 2012; Rastogi et al. 2010). The intent was to utilize materials that are common to indoor decontamination scenarios and cover a range of outcomes (i.e., efficacies), as partial inactivation was desirable for robust comparisons (Rastogi et al. 2010). The four selected materials included industrial carpet (Mannington^®^ Integra HP™, The Home Depot^®^, Cary, NC), latex primed and painted wallboard paper (Georgia Pacific sheetrock facing painted with Painter’s Select^®^ (True Value^®^) Interior PVA drywall primer (PVA-1 white), then Interior Flat finish EZF-1 White Acabado paint), bare structural fir wood (The Home Depot^®^, Cary, NC), and galvanized steel (East Coast Metal, Durham, NC). Each material type was cut into 18 mm diameter discs with thicknesses of ~6 mm (carpet), 0.5 mm (painted wallboard paper), ~5 mm wood, or 0.6 mm (galvanized steel). Each material disc was then affixed to an 18 mm diameter aluminum stub (Ted Pella Inc., Redding, CA) using double-sided carbon tape (Ted Pella Inc., Redding, CA). This combination of material/stub was referred to as a material coupon. All coupons were sterilized by autoclaving (121 °C for 60 min) prior to inoculation and used in the testing described below. Further information on the coupon preparation process can be found in Lee et al. (2011).

### Coupon inoculation

Coupons were inoculated with spores using one of two distinct methods, inoculation with a liquid suspension or via aerosol deposition using the MDI. Regardless of method, the target loading of viable spores was 10^7^ (measured as CFU) per coupon. For liquid inoculation, *B. subtilis* spores were suspended in distilled water at a concentration of 10^8^ CFU/ml and one, 100 µl droplet of this solution was applied to the material surface using a micropipette. The coupons were allowed to dry overnight in a BioSafety Cabinet (BSC); the liquid inoculum remained on the surface as a droplet until the water evaporated (used the same day as inoculation). One exception to the observed drying was the wood coupons, in which the inoculum rapidly soaked into the material.

### Test treatments and controls

For the aerosol-based method, coupons were inoculated using MDIs, as described in Lee et al. (2011). The sterilized coupon was positioned inside the particle deposition chamber. The coupon center and the MDI nozzle were aligned and the distance between the coupon surface and the MDI actuator nozzle was adjusted. After the coupon and MDI were set up, each coupon was inoculated one time by activating the MDI canister. The aerosol-impacted coupon was then immediately removed from the chamber using a sterilized gripper and transported to a circular stainless steel transporting disc. The inoculated coupons were then transported for analysis to the microbiology laboratory.

Four designations of coupons were used in this study: test, positive control, negative control, and blank. Test and positive control coupons were inoculated with spores via one of the two methods described above. Test coupons underwent one of the decontamination methods described below, after their inoculation. Positive controls were not exposed to the decontamination method, however, they were inoculated and extracted along-side the test coupons. Positive controls provided for the determination of viable spore (as CFUs) prior to any decontamination treatment applied to the test coupons. Blank coupons were not inoculated, but they underwent the same decontamination method as the test coupons. The blank coupons were used to indicate any issues related to cross-contamination within this study. For the liquid decontaminant testing, negative control coupons were also used. These coupons were not inoculated nor underwent the decontamination process. The negative control coupons were used to indicate any cross-contamination due to laboratory analysis. The test treatments and replicates per treatment are presented in Tables 1, 2 and 3.

**Table 1:** Test matrix for fumigation ClO_2_ (750 ppmv ClO_2_, 24 °C, 75 % RH)

Test	Material type	Inoculation method	Actual chamber			Contact times (h)	Coupon numbers		
			ClO_2_ concentration (ppmv) (SD)	Temperature (°C) (SD)	% RH (SD)		Positive controls^a^	Test^b^	Field blanks^b^
1	Carpet	Aerosol	688 (21)	23.9 (0.06)	75.2 (0.2)	1, 2, 4, 6, 12	3	3	1
		Liquid					3	3	1
2	Galvanized steel	Aerosol	699 (17)	24.0 (0.11)	77 (0.5)	1, 2, 4, 6, 12	3	3	1
		Liquid					3	3	1
3	Wood	Aerosol	670 (57)	23.2 (0.38)	75.8 (2.6)	1, 2, 4, 6, 12	3	3	1
		Liquid					3	3	1
4	Painted wallboard Paper	Aerosol	698 (24)	23.9 (0.08)	84.2 (0.6)	1, 2, 4, 6, 12	3	3	1
		Liquid					3	3	1

^a^Number of coupons per test

^b^Number of coupons per time point within a test

**Table 2:** Test matrix for the pAB spray tests

Test	Material type	Inoculation method	Contact times (min)	Coupon numbers		
				Positive controls^a^	Test^b^	Field blanks^b^
1A	Galvanized steel	Aerosol	10, 30, 60	5	5	1
1L		Liquid		5	5	1
2A	Carpet	Aerosol	10, 30, 60	5	5	1
2L		Liquid		5	5	1
3A	Wood	Aerosol	10, 30, 60	5	5	1
3L		Liquid		5	5	1
4A	Painted wallboard paper	Aerosol	10, 30, 60	5	5	1
4L		Liquid		5	5	1

^a^Number of coupons per test

^b^Number of coupons per time point within a test

**Table 3:** Test matrix for the pAB immersion tests

Test	Material type	Inoculation method	Contact times (min)	Coupon numbers			
				Positive controls^a^	Test^b^	Negative controls^a^	Field blanks^b^
1	Galvanized steel	Aerosol	1, 15, 20, 30, 60	3	5	3	1
		Liquid		3	5	3	1
2	Carpet	Aerosol	1, 10, 20, 30, 60	3	5	3	1
		Liquid		3	5	3	1
3	Wood	Aerosol	1, 10, 20, 30, 60	3	5	3	1
		Liquid		3	5	3	1
4	Painted wallboard paper	Aerosol	1, 10, 20, 30, 60	3	5	3	1
		Liquid		3	5	3	1

^a^Number of coupons per test

^b^Number of coupons per time point within a test

### Decontamination methods

Three different decontamination methods were used in this study, utilizing two different chemical decontaminants. These decontamination methods were chosen for this study based upon their effectiveness in previous studies, among the many decontaminants considered (Ryan et al. 2010). The first decontamination method (Decontamination Method 1) was fumigation of the coupons with chlorine dioxide gas (ClO_2_). The second and third methods utilized a pH-adjusted bleach solution (pAB) applied to the coupons either by spraying the solution onto the coupon (Decontamination Method 2) or by immersion of the coupon into the solution (Decontamination Method 3).

In Decontamination Method 1, a 730 l glove box (Compact Glove Box 830-ABD, Plas Labs, Inc., Lansing, MI) was covered with foil-backed insulation to make it opaque (because ClO_2_ is light sensitive) and used as the fumigation chamber. The glove box, with anti-chamber, provided a leak-free atmosphere for fumigations, and allowed the periodic addition or removal of coupons during testing. ClO_2_ was generated on-site by a Sabre S07-012 ClO_2_ generator (Sabre Technical Services, LLC, Albany, NY). The S07-012 generates ClO_2_ on a laboratory scale in the same manner as the fumigant is generated for a large-scale fumigation. An aqueous solution of chlorine dioxide is made by mixing hydrochloric acid and sodium chlorite in the presence of aqueous hypochlorite. The chlorine dioxide is then stripped from solution into an air stream moving through a column, thus generating ClO_2_ gas.

ClO_2_ was fed into the fumigation chamber through an actuated valve via polyethylene tubing (1 cm diameter) from the stripping column. The concentration of ClO_2_ was measured in real-time via a ClorDiSys EMS monitor (ClorDiSys Solutions, Inc., Lebanon, NJ) and confirmed every 30 min throughout a fumigation cycle by air sampling and analysis done via adaption of a standard amperometric method (4500-ClO2 Chlorine Dioxide, E. Amperometric Method II 1995). This adapted method for gas sampling for ClO_2_ is described in detail in Wood et al. (2010). Briefly, gas from the chamber is sampled through a series of impingers containing a potassium iodide phosphate buffer (KIPB) solution. The ClO_2_ absorbs and reacts with the potassium iodide. After sampling a predetermined volume of air, the ClO_2_ concentration in the chamber air was determined following titration of the sample with sodium thiosulphate (STS). The real-time concentration measured by the ClorDiSys EMS monitor was used for feedback control, via the actuation of the valve to allow ClO_2_ into the chamber as needed.

The relative humidity (RH) inside the fumigation chamber was also controlled via a feedback loop, with measurement made by the ClorDiSys EMS monitor. The monitor utilized a Vaisala RH/Temperature sensor (model HMD40Y, Vaisala, Helsinki, Finland) to measure RH in real-time. When the RH reading was lower than the RH setpoint, solenoid valves were opened to inject humid air from a gas humidity bottle (LF-HBA, Fuel Cell Technologies, Albuquerque, NM). The gas humidity bottle, heated to 60 °C, passed compressed air through Nafion^®^ tubes surrounded by de-ionized water, creating a warm air stream saturated with water vapor. Similarly, temperature was measured with the Vaisala sensor, and via the ClorDiSys EMS monitor. Temperature control was achieved by fans passing air over temperature-controlled water in radiators. In addition to the three switched fans in operation during heating or cooling, a single fan was always in operation to provide mixing. A pressure relief valve was added to the chamber to prevent over-pressurization (above a set-point); the chamber was maintained under a slight vacuum.

A fumigation cycle consisted of placing the Petri dishes containing the test and blank coupons into the chamber, equilibrating the chamber to the desired temperature and RH for the cycle, introducing ClO_2_ as necessary to achieve and maintain the target concentration (±10 % target) for the duration of the cycle (longest time point, see Table 1), followed by aeration of the chamber to reduce the chamber atmosphere to a non-detectable ClO_2_ concentration. The target temperature and RH were maintained within ±0.5 °C and 10 %, respectively. Sets of coupons in Petri dishes were removed at discrete time points during the fumigation cycle in order to determine decontamination efficacy as a function of exposure duration at each ClO_2_ target concentration. Coupons were aerated inside the air-lock prior to removal from the glove box; this aeration was considered neutralization of the samples following decontamination in accordance with previous ClO_2_ studies (Rogers et al. 2006). All coupons were transferred to the on-site microbiology lab in sealed Petri dishes, for analysis as described in the “Sample extraction and enumeration” section.

Coupons were fumigated with 750 ppmv ClO_2_ for up to 12 h at 24 °C and 75 % RH. Sets of coupons were removed at 1, 2, 4, 6, and 12 h. The test matrix is shown in Table 1. A coupon set consisted of 3 test coupons inoculated by aerosol deposition, three test coupons inoculated via liquid suspension, and one blank coupon. A complete fumigation cycle included five coupon sets (one for each exposure duration); each material type was run as a separate fumigation cycle. Three positive controls from each inoculation method (liquid and aerosol) were included in with each test (cycle).

For Decontamination Methods 2 and 3, pAB was prepared in accordance with the U.S. EPA crisis exemption requirements for use against *B. anthracis* spores. (U.S. EPA 2007) pAB was prepared by mixing one part Clorox^®^ bleach (5–6 % sodium hypochlorite; within 1 year of manufacture), nine parts deionized water, and enough 5 % white distilled vinegar (Great Value Model 35255, Wal-mart, Bentonville, Arkansas) to yield a solution having a mean pH of 6.8 and a mean total chlorine content of 6,000–6,700 ppm. Concentration was determined using a Hach CN-HRDT Hypochlorite test kit (Hach Company, Loveland, CO), which uses Hach iodometric Method 10100. An Oakton Acorn pH5 meter was used to measure the acidity of the solution.

**Fig. 1:**
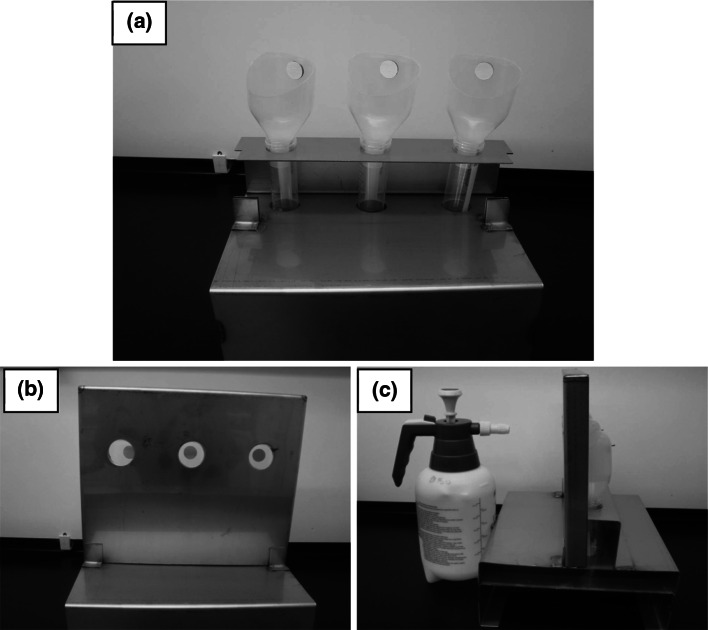
Experimental setup for the pAB spray testing. **a** Sample tubes with modified funnels holding sample coupons. **b** Front and **c** side views of the stage holding three coupons. Despite parallax, coupons were mounted such that they were in the radial center of the opening in the spray guard

For Decontamination Method 2 (spray application of pAB), each test or blank coupon was attached to a specially designed funnel that was connected to a conical vial to retain the runoff generated during the spraying (see Fig. 1). The coupon assemblies were mounted onto a stage that accommodated three assemblies (see Fig. 1). For each material type, inoculation method and contact time, two stages were used together holding a combined five replicate test coupons and one blank coupon. On each stage, a spray guard was placed approximately one inch in front of the coupon surfaces. The spray guard contained 2.5 cm holes aligned directly in front of each coupon, and allowed the spray to impact on the intended coupon surface while avoiding inadvertent spray to the nearest neighbor. All materials were sterilized between tests, prior to use, by autoclaving (121 °C for 60 min).

An RL Flo-Master^®^ Premium Home and Garden 1/2 gallon sprayer (Model 1101HD, Rool-Lowell Manufacturing, Inc, Lowell, MI) was used at its initial (lowest) nozzle settings to achieve the finest (i.e., droplet size) spray. Prior to use, the spray bottles and nozzles were disinfected by rinsing them once with pAB and then three times with sterile deionized water (DW). A spray bottle containing pH-amended bleach solution was used to spray the test coupons; a second spray bottle filled with DW was used to spray the blank coupon. During spraying, the spray nozzles were maintained approximately six inches from the surface of the coupon and horizontally aligned with the center of the coupon. During the decontamination process, the coupons were sprayed at time intervals necessary to maintain a wetted look up to the desired contact time. Galvanized steel, wood, and painted wallboard paper were sprayed initially and at 3 min intervals while carpet required 4 min intervals, for the duration of the contact time. After the desired contact time, each coupon was aseptically transferred to a sample tube containing 10 ml BBL™ buffered peptone water (BD™; Becton, Dickinson, and Company; Franklin Lakes, NJ) containing 0.01 % Tween-80™ (Fisher Scientific, Pittsburgh, PA)10 ml of buffered peptone water (BPW) (make model, manufacturer, location). Each funnel was rinsed with 20 ml of DW and 10 ml of BPW was then added to the rinsate. All samples were capped and transferred to the on-site microbiology lab for analysis as described in the “Sample extraction and enumeration” section.

For Decontamination Method 3 (immersion in pAB), test coupons were aseptically transferred to individual 50 ml sterile centrifuge tubes into which 10 ml of pAB was added to each using a sterile serological pipette. The coupons remained submerged in the pAB for the desired contact time, after which point the solution was neutralized with the addition of 10 ml of a sterile STS solution to each centrifuge tube. The solution from each tube was then decanted into separate sterile 50 ml sample tubes, and retained. BPW (10 ml) was then added to each tube containing the coupon. All samples (centrifuge tubes with coupons and decanted, neutralized pAB) were capped and transferred to the on-site microbiology lab for analysis as described in the “Sample extraction and enumeration” section. The matrices of tests involving pAB can be found in Tables 2 (spray) and 3 (immersion).

### Sample extraction and enumeration

As described in Lee et al. (2011), coupons from Decontamination Methods 1 testing were aseptically removed from the Petri dishes and placed in 50 ml sterile vials with 10 ml BPW with Tween 80. For Decontamination Methods 2 and 3, coupons were already in sterile vials in the buffer solution (see above). To extract the spores from the coupon surface, coupons were subjected to a 10 min sonication (Ultrasonic Cleaner FS140, Fisher Scientific, Pittsburg, PA) followed by 2 min vortexing (Mini Vortexer 128,101, Fisher Scientific, Pittsburg PA). Ten-fold serial dilutions were then prepared for each sample, as needed, by adding 0.1 ml of the aqueous buffer from the sample to 0.9 ml of BPW using a micropipette. Appropriate dilutions were spread in triplicate (0.1 ml each) onto TSA (BD™; Becton, Dickinson, and Company; Franklin Lakes, NJ) plates and incubated at 35 ± 2 °C for approximately 18 h. Colonies consistent with the morphology of the target organism were then counted manually for all plates. Results (CFU) were reported for all plates having between 0 and 300 colonies. The surface spore concentration (CFU/coupon) reported for each sample was determined by averaging the results from triplicate subsamples and calculating per Eq. (1).

1 $$ \frac{CFU}{coupon} = \frac{P \times V}{I \times D} $$

In Eq. (1), P is the average of the CFU on the triplicate plates, I is the volume of sample added to each plate (0.1 ml), D is the tube dilution factor, and V is the total volume of liquid in the sample extract (typically, 10 ml in this study as described above). The initial tube containing the 10 ml of extraction solution is considered the zero dilution (10°). Therefore, as an example, an average of 100 CFU on the triplicate plates determined from the third dilution (10^−3^) would equate to 1.0 × 10^7^ CFU/coupon. At the lowest limit of observable growth, 1 CFU on one of three triplicate plates at the zero dilution would equate to a mean value of 33 CFU/coupon. While this mean value is the lowest non-zero average spore recovery that can be determined from the current method, it is not the method detection limit (MDL) since it does not account for the probability of detection due to sub-sampling (e.g., sampling a total of 0.3 ml out of 10 ml).

When no viable spores were detected on a plate, a value of 0.5 CFU (one-half the quantitation limit of detection, 1 CFU) was substituted for zero (i.e., 0.5 CFU/sample (coupon)). The substitution of the quantitation limit (or one-half this limit) is consistent with the treatment of non-detects in similar published work (U.S. EPA 2011b; Rastogi et al. 2009, 2010; U.S. EPA 2010).

### Data analysis

For statistical analysis, the data were fit to an exponential model (Eq. (2)) of the form:

2 $$ \log_{10} (y/y_{o} ) = {\text{a}}(1 - e^{{b{\text{CT}}}} ) $$

Here, y is the average number of spores on each plate (in CFUs) from test coupon extracts, y_o_ is the average number of spores (CFU) recovered from the corresponding positive control coupon extracts, *a* is the multiplicative term used for scaling, *b* is a term indicating the rate of decontamination, C is the concentration of ClO_2_ (in ppmv), and T is the time (in hours) the coupon was in contact with the decontaminant. For the pAB data, C was set to unity since concentration was not a parameter in this part of this study. The resulting fit parameters (*a*, *b*) for each combination of inoculation method, material type, and decontamination method (ClO_2_, pAB spray, and pAB immersion) were determined (using Origin^®^ 7, OriginLab^®^, Northampton, MA), including the best-fit values, approximate error, and 95 % confidence interval. These data were used to test for statistically significant differences due to variations in study parameters, at the 95 % confidence level. This model was used to provide a best empirical fit to the data and not to imply understanding of the inactivation kinetics.

Expressing the data as log_10_ (y/y_o_), as in Eq. (2), is consistent with the reporting of log reduction (LR) as done in other relevant efficacy studies (U.S. EPA 2010, 2011b; Rastogi et al. 2009, 2010; Calfee et al. 2011; Wood et al. 2011). However, in these citations, LR is typically reported as the inverse (i.e., log_10_ (y_o_/y)). In both cases, the magnitude of the reported value is the equivalent and can be compared across studies.

Direct comparisons of average values, e.g., positive controls or LR values from two test groups, were performed where discussed using the Student’s t Test. An unpaired test with a confidence interval of 0.05 (95 %) was used to calculate statistical probabilities. A two-tail *p*_value_ was used to indicate the chance that randomly selected samples could have means at least as far apart as observed if the null hypothesis were to be true. The null hypothesis was that there was no difference in the means of the test groups; i.e., the means are likely from the same population. It should be noted that although a small *p*_value_ (i.e., <0.05) may suggest that the null hypothesis is false, other factors may also contribute. The evidence should not automatically be taken to disprove the truth of the hypothesis. For example, 95 % confidence intervals lying entirely within the range of indifference may tend to provide further support that the means were truly different to experimental parameters (e.g., inoculation method).

## Results

The average recoveries for all positive controls used in this study are reported in Table 4, as a function of inoculation method and material type.

**Table 4:** Average CFU and residual standard deviation (RSD) from positive controls

Material type	Inoculation method	Average CFU	RSD (%)	Number of replicates
Galvanized steel	Aerosol	3.46E+07	36	10
	Liquid	2.50E+07	29	9
Carpet	Aerosol	6.84E+07	61	11
	Liquid	3.84E+07	41	11
Wood	Aerosol	1.04E+08	25	11
	Liquid	2.66E+07	59	11
Painted wallboard paper	Aerosol	8.22E+06	34	9
	Liquid	4.83E+07	27	10

For the fumigation cycles, the actual chamber ClO_2_ concentration, temperature, and RH for the four tests are shown in Table 1. For the spray and immersion tests, the pAB was determined to be within the target pH and chlorine content ranges prior to use in all tests. The pAB solution was prepared fresh on each day of testing and used within 3 h of preparation.

None of the negative control and blank coupons used throughout this study indicated any evidence of cross-contamination. No viable target organism was recovered from any of the blanks or negative controls.

**Fig. 2:**
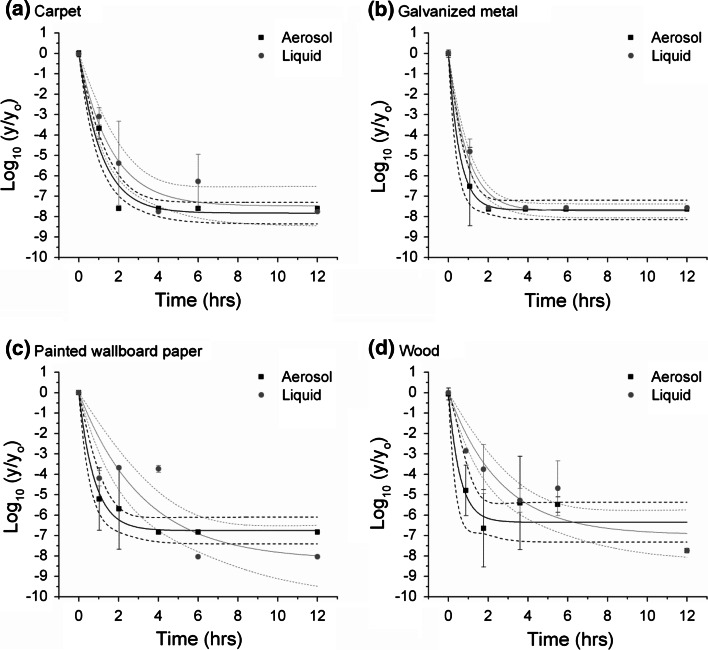
Log reduction in viable spores as a function of chlorine dioxide fumigation time. *Plots* show the average and standard deviation of the log of the measured CFU values at each time point on the test coupons (y) divided by the average of the positive controls (yo). The fits to the data and 95 % confidence intervals are also shown. Aerosol inoculation is in *black* and liquid inoculation is in *gray*

**Fig. 3:**
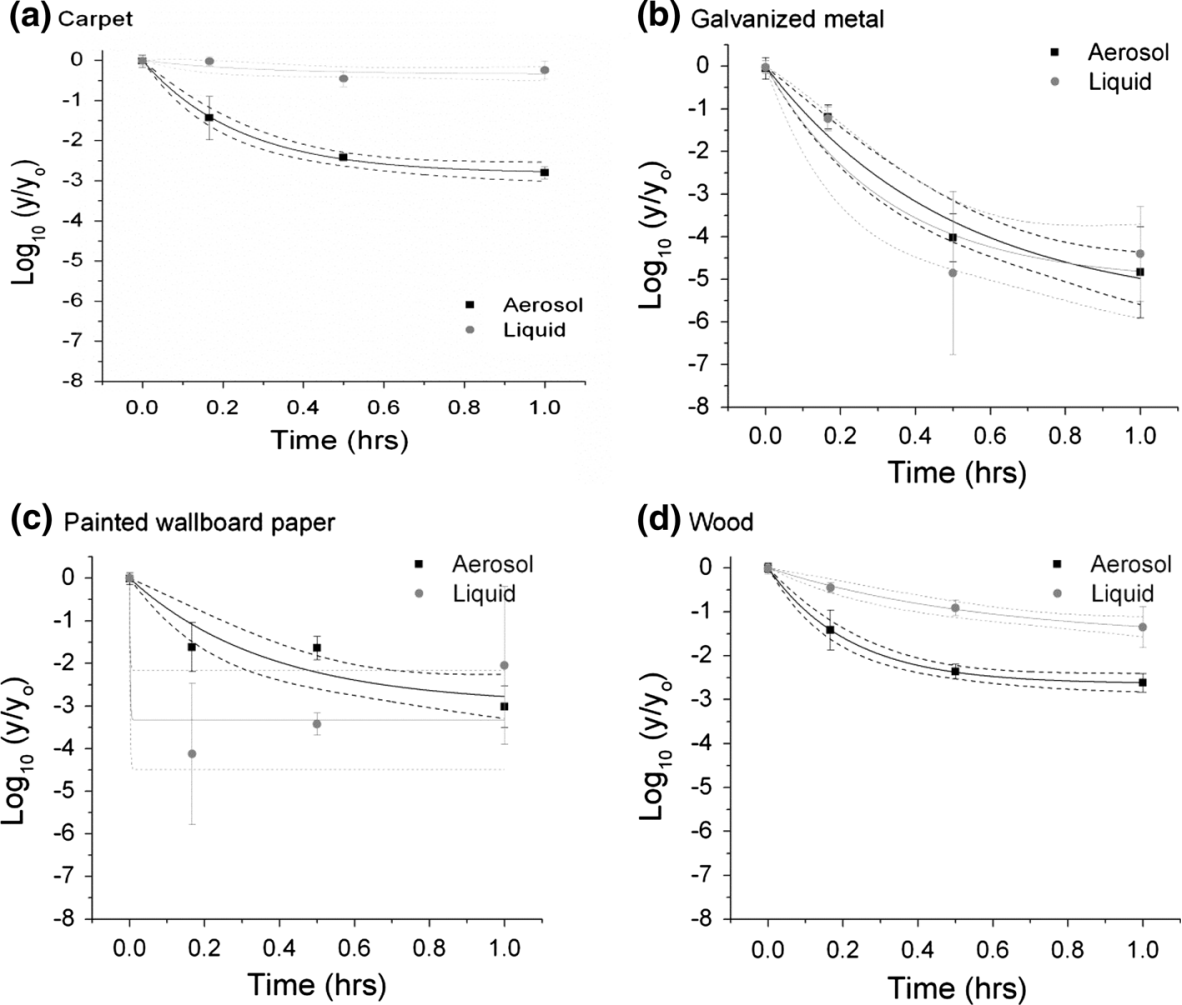
Log reduction in viable spores as a function of time for spraying with pH-adjusted bleach. *Plots* show the average and standard deviation of the log of the measured CFU values at each time point on the test coupons (y) divided by the average of the positive controls (yo). The fits to the data and 95 % confidence intervals are also show. Aerosol inoculation is in *black* and liquid inoculation is in *gray*

**Fig. 4:**
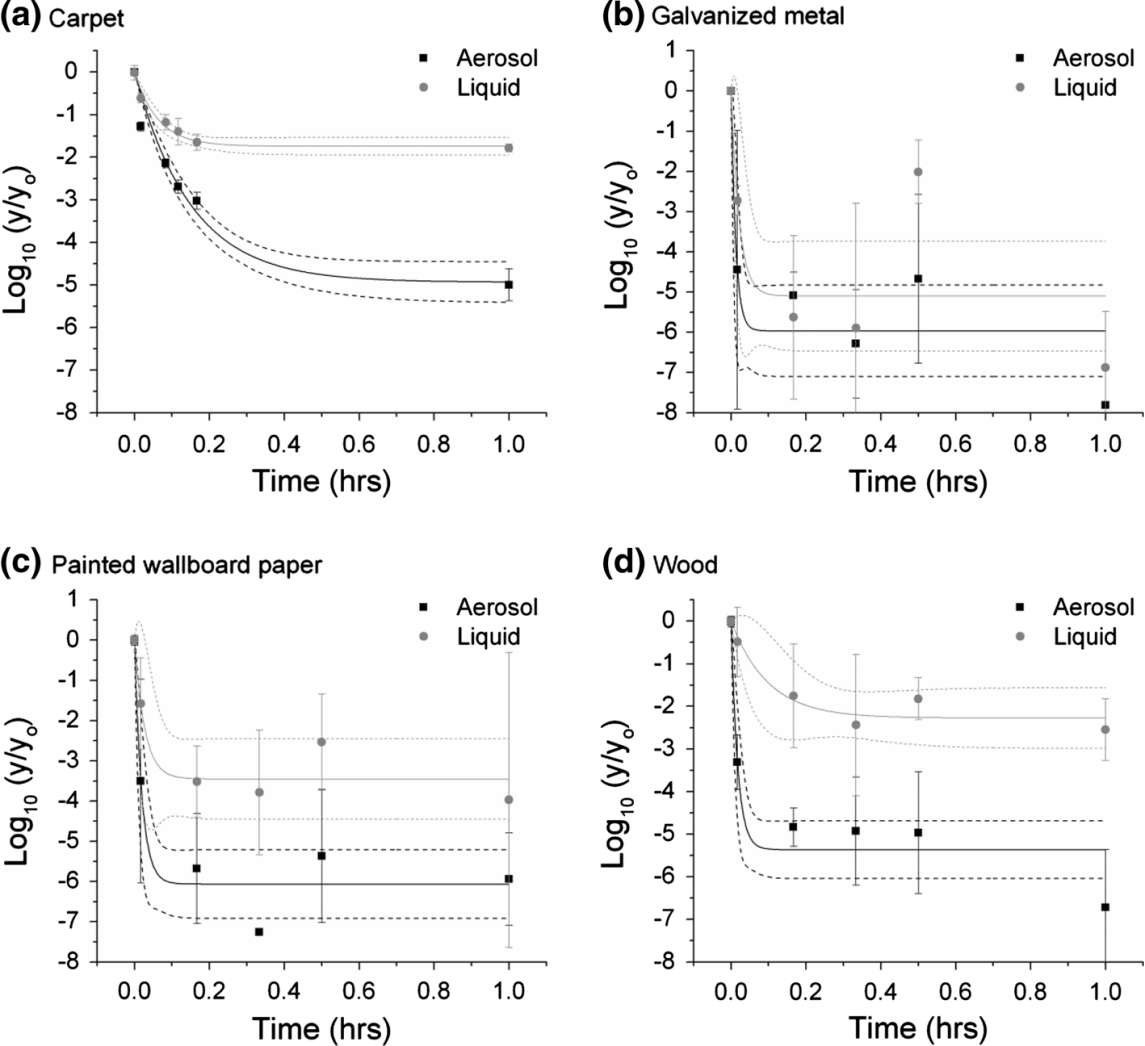
Log reduction in viable spores as a function of time for immersion in pH-adjusted bleach. *Plots* show the average and standard deviation of the log of the measured CFU values at each time point on the test coupons (y) divided by the average of the positive controls (yo). The fits to the data and 95 % confidence intervals are also show. Aerosol inoculation is in *black* and liquid inoculation is in *gray*

The reduction in viable spores recovered from the liquid- and aerosol-inoculated test coupons was evaluated as a function of exposure time for fumigation with ClO_2_, spraying with pAB, or immersion in pAB. The average log of the reduction (with standard deviation) is plotted as a function of time for each of the decontamination treatments in Figs. 2, 3 and 4. Additionally, the data fits using Eq. (2) and the 95 % confidence intervals are plotted. Where confidence intervals do not overlap, significant difference in the resulting fits is suggested. The rate of decontamination can be observed in Figs. 2, 3 and 4 as the increasing magnitude of LR as a function of time.

Based upon statistical analysis of fit comparisons, no impact of the inoculation method on the overall log reduction was observed for fumigation with ClO_2_ under the conditions used in this test (i.e., at the 12 h time point). A greater than 6 LR was observed for all materials by 6 h of fumigation at 750 ppmv (75 % RH and 24 °C). In all cases, no detectable CFUs were recovered from any test coupons by at least the maximum fumigation time (12 h) for all four material types. However, the actual time required to achieve that 6 LR was a function of the material type, and (depending upon the material) inoculation method.

In general, the rate of decontamination was lower for the liquid inoculated coupons compared to the aerosol inoculation on the same material type. However, the difference was not statistically significant for all material types. Galvanized metal was the easiest to decontaminate, as noted by the most rapid decrease in recovered viable spores compared to the positive controls for that material (i.e., decrease in Log_10_ (y/y_o_)). Wood was the most difficult. For liquid inoculation, the order of increasing difficulty in decontamination was: galvanized metal, painted wallboard paper, carpet, and wood. For aerosol inoculation, the order of increasing difficulty (slower rate of decontamination) was: galvanized metal, painted wallboard paper ≈ carpet ≈ wood. A statistically significant difference in the rate of decontamination was not observed for the latter three materials. For painted wallboard paper and wood, the rate of decontamination was significantly slower for liquid- compared to aerosol-inoculated coupon sets. Although the rate for the liquid inoculated coupon sets was also estimated to be less than that of aerosol-inoculated sets for carpet and galvanized metal, this difference was not quite statistically significant.

Similarly, during the pAB spray-based tests, the liquid-inoculated coupon sets were more difficult to decontaminate than their aerosol counterparts (see Fig. 3) for some materials based upon comparison of decontamination rates and overall log reductions. The difference was greatest for carpet and wood, the two most difficult to decontaminate materials by this method. No observed difference in the rate of decontamination as a function of inoculation method was observed on galvanized metal, the easiest material to decontaminate using pAB spraying. The model did not fit well for the liquid inoculated painted wallboard paper; this was due to the wide variability observed at each time point for this coupon set/decontaminant combination. In all cases, regardless of material type or inoculation method, a 6 LR was not achieved within a 1 h contact time (with repeated decontaminant applications as noted in the “Materials and methods” section).

Immersion in pAB resulted in significantly greater magnitude log reduction values for both liquid and aerosol inoculation compared to the results from the spray testing (see Fig. 4). This was true for all materials and both inoculation methods. Statistically significant differences in efficacy were noted for liquid versus aerosol inoculation for carpet, painted wallboard paper, and wood coupon sets. Decontamination of three of the four materials was significantly more difficult for the liquid inoculated coupons both in terms of an observed slower rate of decontamination and the final log reduction values, However, no observable, statistically significant, difference with respect to the impact of inoculation method was detected for galvanized metal; this material was again among the easiest to decontaminate. In the case of the other three materials, the inoculation method had a dramatic impact on the efficacy. The magnitude of the log reduction measured by the aerosol inoculation method was at least 5, and reached 6 for painted wallboard paper. Alternatively, for the liquid inoculated coupon sets, the magnitude of the log reduction was less than 4 for painted wallboard paper and less than 2 for both carpet and wood.

## Discussion

The target spore loading in this study was at least 10^7^ CFU (7 logs) per coupon with a relative standard deviation (RSD) of less than 50 % for each material type. This target loading (Table 4), based upon the analysis of the positive controls, allowed for a greater than 6 LR to be determined with respect to the effectiveness of the decontamination parameters. A 6 LR has been used as a standard for the definition of an effective decontaminant against *B. anthracis* (U.S. Environmental Protection Agency FIFRA SAP Meeting No. 2007-50  2007; Ryan et al. 2010; U.S. EPA 2010); hence, test protocols meant to produce data with respect to the quantitative effectiveness of decontamination methods should have at least a 6 log dynamic range.

Consistent with the findings reported by Lee et al. (2011), the results from the positive controls from all coupons inoculated via aerosol deposition achieved the target loading (see Table 4). With the exception of carpet, the RSD was less than the target of 50 % for all other aerosol-inoculated coupon types. The RSD criteria was set at 50 % for the aerosol inoculation method development (refer to Lee et al. 2011) in order to be comparable to the RSD typically observed with liquid inoculation. Comparable RSDs for liquid and aerosol inoculation methods can be observed from the data set. For galvanized metal and painted wallboard paper, the RSD values are quite comparable. A ~20 % difference is noted for carpet and wood. The aerosol method resulted in a lower RSD on wood, while the liquid method was more consistent on carpet. However, both methods resulted in greater than 6-log CFU recoverable from the surfaces and RSD were factored in during the statistical analysis, i.e., the determination of the impact of decontamination method parameters in the partial-inactivation regimes.

Recoveries from carpet, painted wallboard paper, and wood positive control samples varied significantly by inoculation method. For carpet and wood, the recoveries from the positive controls were greater from inoculation with aerosol than that from liquid-inoculated samples. The difference was greatest for wood, with recovered CFU after aerosol inoculation being nearly a full log higher than liquid-inoculated samples. As discussed in the previous study by Lee et al. (2011), the spores by liquid inoculation may be transported through the wood crevices while the liquid inoculum soaked into the wood surface. The spores by aerosol inoculation method are deposited by impaction to the wood surfaces, so the penetration of spores through the wood crevices is limited. The spores by aerosol inoculation are mostly deposited near the top of the wood surface and are relatively easy to be extracted compared to the liquid inoculation. For painted wallboard, the average recovered loading was higher with liquid inoculation. Per the surface analysis reported in Lee et al. (2011), the deposition pattern on the painted wallboard coupons that were liquid inoculated showed spore bands at the outer edge of the inoculated area. This was consistent with spore migration to the outer edge of the droplet during the water evaporation. For the aerosol inoculation method, deposition created a center-crowded pattern produced by the spray action from the MDI actuator nozzle. Although the mechanism is unclear based upon this information alone, this noted variance may contribute to the observed difference in recovery. However, although similarly different deposition patterns were observed for galvanized metal based upon the inoculation method, no statistically significant difference in recovered spore loading was evident.

The difference in recovered loading can be attributed to many complex factors that are difficult, if not impractical, to control in such a study. For example, recovery efficiency of *Bacillus* spores can vary greatly as a function of material substrate type (Rastogi et al. 2009; Calfee et al. 2011; Wood et al. 2011). All of these cited studies used liquid inoculation. These results were expected and consistent with the finding reported by Edmonds et al. (Edmonds et al. 2009), using a similar aerosol deposition method. The authors concluded that recovery of spores differed by inoculation method and as a function of material type; in some cases recovery of liquid-inoculated spores was higher than aerosol-deposited spores, and vice versa. As discussed above, the interaction of the liquid with the substrate can be one factor that impacts the distribution of the spores on the coupon and, hence, the recovery from the material via the chosen sampling method (here, liquid extraction). Addressing the challenges associated with the recovery of spores from materials was well beyond the scope of this study. In addition, the resulting differences in observed starting loading (as determined from the positive controls) could confound such conclusions from the current study. Therefore, during statistical analyses, the data were normalized to the positive control values to assess the impact of inoculation method on decontamination effectiveness.

Testing with aerosolized *B. anthracis* Ames presents significant biological safety concerns. Hence, the use of appropriate non-pathogenic surrogates is necessary to conduct many of the applied studies without the safety restrictions necessitated when using fully virulent *B. anthracis*. As concluded by Spotts Whitney et al. (2003) surrogates do not always adequately predict the behavior of the target species. Appropriate surrogates for decontamination efficacy testing can be highly dependent upon the decontaminant being tested. For example, conclusions drawn from numerous test data have indicated that *B. subtilis* (consistent with the preparation used in this work) can appropriately represent *B. anthracis* with respect to decontamination efficacy testing using fumigation with ClO_2_ or inactivation with pAB (Tomasino et al. 2008; U.S. EPA 2011b). However, this is not the case for methyl bromide, as *B. subtilis* is significantly more resistant to this decontaminant than *B. anthracis* Ames (U.S. EPA 2011b).

The observed exponential decay of efficacy (LR) with increasing decontamination time suggests a slowing decontamination rate (or tailing off of the spore inactivation), and is common with quantitative sporicidal efficacy testing. As reported by Ryan and Rastogi (U. S. Environmental Protection Agency 2008), inactivation of *B. anthracis* spores on materials by fumigation with ClO_2_ has been observed to follow non-linear kinetics. The rate of inactivation is initially fast and slows with increasing time. This phenomenon seems consistent with other investigations of microbial persistence and inactivation, as non-linear responses are often attributed to the presence of a highly resistant sub-population of cells (Withell 1942) or to the rate-lessening effects of substrate (viable cells) limitation over time (Chick 1910). The kinetics and overall decontamination efficacy have been observed to be strongly dependent on material type (U. S. Environmental Protection Agency 2008; Wood 2009; Rastogi et al. 2010; U.S. EPA 2011b). Bare wood has been one of the most difficult materials to decontaminate (Wood et al. 2011; Calfee et al. 2011; Rastogi et al. 2010). Due to this dependency, multiple materials with differing degrees in anticipated challenge for decontamination with ClO_2_ and pAB were selected for this study. If materials that are too easy to decontaminate were used, the decontamination rate may proceed too fast for an impact of the test parameters (e.g., inoculation method, material type, decontamination method) to be measured. Conversely, rates that are too slow due to overly challenging materials may also mask any dependence of the efficacy on the test parameters. Therefore, materials exhibiting a range of anticipated decontamination challenge are essential in such a study in order to draw relevant and applicable conclusions.

A statistical comparison of the data for fumigation with ClO_2_ suggests that liquid inoculation can be an increased challenge to decontamination efficacy testing when compared to the aerosol inoculation method used in this study (see Fig. 2); this increased challenge is most effectively observed for the materials that are more difficult to decontaminate. However, for fumigation with ClO_2_ at the conditions used in this study, liquid inoculation did not significantly under-represent the observed effectiveness of the method nor the determination of effective (sporicidal) conditions. At these test conditions, also used for past Amerithrax and subsequent fumigations (Martin 2003; U. S. Environmental Protection Agency 2005), the difference was not practically relevant.

For decontamination using pAB spraying, the results suggest that liquid inoculation could potentially result in an underestimate of the effectiveness when compared to use of an aerosol inoculation method (see Fig. 3). This was most apparent for carpet, followed by wood (no significant difference for the other two materials). For example, very little to no log reduction was observed on carpet that was liquid-inoculated. A 2.8 (±0.14) average (standard deviation) log reduction was measured for the aerosol inoculated carpet coupon set. While this is a large difference in relative effectiveness, it did not result in the under-reporting of overall effectiveness. In other words, spraying with pAB at the conditions tested did not achieve the 6 LR laboratory testing target that has been reportedly achieved by other sporicidal decontamination methods (e.g., such as ClO_2_ in this study) (U.S. EPA 2011b; Rogers et al. 2006, 2007; Rastogi et al. 2009, 2010).

Similarly for the immersion in pAB, liquid inoculated coupons were more challenging to decontaminate than their corresponding aerosol-inoculated counterparts. This was the case for three of the four material types, the exception being galvanized metal. Galvanized metal was the easiest material to decontaminate, hence the decontamination rate was too fast regardless of inoculation method to be able to resolve a difference in the current study.

Overall, the statistically significant differences observed for the harder to decontaminate materials (those with a slower decontamination rates, i.e., longer times required to achieve a 6 LR) across all three decontamination methods suggests that tests utilizing liquid inoculation can potentially under-predict the rate of the decontamination and overall effectiveness compared to inoculation using an aerosol method. However, the practical importance highly depends upon the material, decontaminant product, test method, and purpose of the testing. Therefore, based upon these findings, inoculation method is one factor to consider when devising efficacy studies, but its relative importance compared to other test parameters needs to consider the practical data requirements.

By way of example for pAB, the impact of inoculation methods on efficacy determination was not nearly as substantial an influence as that of the liquid decontaminant application method. Immersion in pAB resulted in significantly higher reductions in the number of recoverable viable spores from the test coupons compared to maintaining the materials wetted by pAB spraying. This result holds true for all four material and both inoculation methods (compare Figs. 3, 4). Based upon these results, the decontaminant application method (immersion vs. spraying) had a more pronounced effect on the measured effectiveness of the product than did the inoculation protocol. Results suggest that immersion testing could dramatically over-indicate the sporicidal ability of the product when it is used as a spray. These findings draw into question the use of immersion-based testing for predicting decontaminant performance on complex surfaces. While immersion tests are desirable for their repeatability, spray-based methods may offer a more operationally-relevant understanding of its ability to be deployed for field-use. Challenges such as application evenness and having a low ratio of decontaminant to contaminated surface area are common to both field-use and spray-based efficacy test methods. In addition, evidence suggests that the measured effectiveness can be influenced by spray parameters (e.g., flowrate, pressure, duration, frequency) (Ryan et al. 2012; Calfee 2012). Although the application procedure for this study was designed to keep the materials wetted, the results may not be indicative of a procedure using different spray parameters. The immersion versus spray results for pAB highly suggests that the end application procedure of the decontaminant be thoroughly considered when developing specific efficacy test method procedures or parameters.

The data used for the analysis described above used one-half the quantitation limit for non-detects (0.5 CFU), as discussed in the “Materials and methods” section. For comparison, the method detection limit (MDL) was determined from the experimental results as the one-sigma MDL in accordance with US EPA 40 CFR (U.S. EPA 2011a). The MDL was determined by calculating the average standard deviation of measurements near the detection limit, and modifying this calculated value by the appropriate t-score for the desired confidence limit (one-sigma, 68 %) and degrees of freedom (≈5). Assuming homogeneity between the measurements for each data set and that the data used for this analysis are near the MDL, the MDL was determined to be 101 CFU (204 CFU at 95 % confidence, or 341 CFU at 99 % confidence) per sample (coupon). This MDL was not determined to be statistically significantly different for the two inoculation methods. The use of this MDL to replace all non-detects did not result in any differences with respect to the impact of inoculation method (compared to when 0.5 CFU/coupon was used). Using the MDL value did truncate the dynamic range of the test method, i.e., reducing the range by 2 logs, hence, the maximum quantifiable magnitude of the log reduction when considering this MDL (101 CFU) and starting with a 7 log inoculation target is 5. This is a full log less than the target value of a 6 LR used as the measured of a sporicidal decontaminant in laboratory-scale efficacy testing.

It should be noted that the MDL determined statistically post hoc from the data generated in this study is consistent with the values report by Brown et al. (2007) in their sampling studies. For wipe sampling, Brown et al. (2007) reported quantitative limits of detection per unit of sampled area of 90 CFU for stainless steel and 105 CFU for painted wallboard. For vacuum sampling, Brown et al. (2007) reported quantitative limits of detection per unit of sampled area of 105 CFU for stainless steel and carpet, 120 CFU for painted wallboard, and 160 CFU from bare concrete. The detection limits reported in the cited work are for the combination of the surface sampling, extraction of the sampling media and spread plate culturing and counting. These MDLs are consistent with those found in our work using coupon extract, spread plating, culturing, and counting. The importance here is that the results of the small scale laboratory testing are consistent with more application-related studies (e.g., utilizing field sampling procedures rather than material extraction). While larger scale studies incorporating field methodologies may offer some advantages related to ensuring efficacy testing relates to field use (e.g., immersion vs. spray testing), it should be pointed out that smaller scale studies are not irrelevant based upon some perceived methodology differences. Smaller scale studies using standardized laboratory procedures, e.g., coupon extraction, may offer more control of confounding factors. However, an important aspect of this discussion for future consideration is the impact of not using an actual MDL on the artificial expansion of the dynamic range of an efficacy test method.

Based upon this work and the cited literature, it is clear that inoculation method, decontaminant application method, and treatment of detection limits can have an impact on the sporicidal efficacy measurements. The relative importance of each of these factors with respect to each other and other test parameters is highly dependent upon the ultimate intended use of the data, i.e., the practical application. Each parameter should be considered with respect to its impact on the anticipated data during study design. The results of this work are intended to aid in the consideration of sporicidal efficacy test data when determining which decontamination options might be appropriate options based upon site specific needs and intended use.
